# Long Persistence of a *Streptococcus pneumoniae* 23F Clone in a Cystic Fibrosis Patient

**DOI:** 10.1128/mSphere.00201-17

**Published:** 2017-06-07

**Authors:** Martin Rieger, Harald Mauch, Regine Hakenbeck

**Affiliations:** a Department of Microbiology, University of Kaiserslautern, Kaiserslautern, Germany; b HELIOS Klinikum Emil Von Behring, Berlin, Germany; JMI Laboratories

**Keywords:** 23F clone, *Streptococcus pneumoniae*, cystic fibrosis, hyaluronidase, penicillin-binding proteins, persistence

## Abstract

*Streptococcus pneumoniae* isolates of serotype 23F with intermediate penicillin resistance were recovered on seven occasions over a period of 37 months from a cystic fibrosis patient in Berlin. All isolates expressed the same multilocus sequence type (ST), ST10523. The genome sequences of the first and last isolates, D122 and D141, revealed the absence of two phage-related gene clusters compared to the genome of another ST10523 strain, D219, isolated earlier at a different place in Germany. Genomes of all three strains carried the same novel mosaic penicillin-binding protein (PBP) genes, *pbp2x*, *pbp2b*, and *pbp1a*; these genes were distinct from those of other penicillin-resistant *S. pneumoniae* strains except for *pbp1a* of a Romanian *S. pneumoniae* isolate. All PBPs contained mutations that have been associated with the penicillin resistance phenotype. Most interestingly, a mosaic block identical to an internal *pbp2x* sequence of ST10523 was present in *pbp2x* of *Streptococcus mitis* strain B93-4, which was isolated from the same patient. This suggests interspecies gene transfer from *S. pneumoniae* to *S. mitis* within the host. Nearly all genes expressing surface proteins, which represent major virulence factors of *S. pneumoniae* and are typical for this species, were present in the genome of ST10523. One exception was the hyaluronidase gene *hlyA*, which contained a 12-nucleotide deletion within the promoter region and an internal stop codon. The lack of a functional hyaluronidase might contribute to the ability to persist in the host for an unusually long period of time.

**IMPORTANCE** *Streptococcus pneumoniae* is a common resident in the human nasopharynx. However, carriage can result in severe diseases due to a unique repertoire of pathogenicity factors that are rare in closely related commensal streptococci. We investigated a penicillin-resistant* S. pneumoniae* clone of serotype 23F isolated from a cystic fibrosis patient on multiple occasions over an unusually long period of over 3 years that was present without causing disease. Genome comparisons revealed an apparent nonfunctional pneumococcus-specific gene encoding a hyaluronidase, supporting the view that this enzyme adds to the virulence potential of the bacterium. The 23F clone harbored unique mosaic genes encoding penicillin resistance determinants, the product of horizontal gene transfer involving the commensal *S. mitis* as donor species. Sequences identical to one such mosaic gene were identified in an *S. mitis* strain from the same patient, suggesting that in this case *S. pneumoniae* played the role of donor.

## Abstract (precis)

*Streptococcus pneumoniae* is a common resident in the human nasopharynx. However, carriage can result in severe diseases due to a unique repertoire of pathogenicity factors that are rare in closely related commensal streptococci. We investigated a penicillin-resistant* S. pneumoniae* clone of serotype 23F isolated from a cystic fibrosis patient on multiple occasions over an unusually long period of over 3 years that was present without causing disease. Genome comparisons revealed an apparent nonfunctional pneumococcus-specific gene encoding a hyaluronidase, supporting the view that this enzyme adds to the virulence potential of the bacterium. The 23F clone harbored unique mosaic genes encoding penicillin resistance determinants, the product of horizontal gene transfer involving the commensal *S. mitis* as donor species. Sequences identical to one such mosaic gene were identified in an *S. mitis* strain from the same patient, suggesting that in this case *S. pneumoniae* played the role of donor.

## INTRODUCTION

*Streptococcus pneumoniae* is a common member of the commensal flora of the nasopharynx, particularly in children. Carriage rates between 5% and 20% have been observed in healthy children in Europe and the United States (1–3); however, high rates of over 80% are reported occasionally (4–6) and especially in developing countries (7, 8). Carriage may lead to a variety of diseases, such as otitis media, pneumonia, septicemia, and meningitis, especially in young children, elderly people, and immunocompromised patients (for a review, see reference 9). In fact, pneumococcal infections cause more deaths than other infectious diseases worldwide. The pathogenic potential distinguishes *S. pneumoniae* from other members of the group of viridans streptococci (10).

Numerous virulence factors of *S. pneumoniae* have been described (for a review, see references 11 and 12), but most of them are also present in the closely related commensal *Streptococcus* species *S. mitis*, *S. pseudopneumoniae*, and *S. oralis* (13–15). Typical for *S. pneumoniae* is the polysaccharide capsule, which is crucial for the pathogenicity of this species. Over 90 capsular types have been reported, based on biochemical and genetic analyses (16), and the potential to cause disease depends on the serotype (ST) (17). Before introduction of pneumococcal conjugate vaccines (PCVs), only a few serotypes accounted for the majority of invasive diseases, including types 4, 6B, 9V, 14, 18C, 19F, and 23F. The prevalence of serotypes changed after introduction of the first seven-valent conjugated vaccine (PCV7) in 2000, which covered serotypes 4, 6B, 9V, 14, 18C, 19F, and 23F, followed later by PSV10, which also included serotypes 1, 5, and 7F, and by PCV13, with the additional serotypes 3, 6A, and 19A. Vaccination was accompanied by the appearance of antibiotic-resistant clones expressing nonvaccine serotypes (18). Other important virulence factors present in most *S. pneumoniae* strains are the pneumolysin Ply, choline-binding proteins (CBPs), which include the autolysin LytA as well as the variable CBPs PspA, PspC, and PspA, and the hyaluronidase HlyA (11, 12).

Due to its ability for genetic transformation, the genomes of *S. pneumoniae* isolates are highly diverse and include a large accessory genome. The increasing number of available genome sequences has provided an insight into the astounding repertoire of genes available in the pan-genome of pneumococcus (19, 20). The current standard for the definition of clones is based on comparative sequence analysis of housekeeping genes, which are part of the core genome common to all strains of the species; the methods is termed multilocus sequence typing (MLST) (21). The *Streptococcus pneumoniae* MLST database (https://pubmlst.org/spneumoniae) listed 13,126 STs in February 2017. Different capsular serotypes may be found within one ST due to capsule switching (22, 23). Genomes of an identical ST may vary considerably in their accessory genome content (24).

We report here on a rare clone of serotype 23F *S. pneumoniae* representing isolates with intermediate penicillin resistance which have been collected over a period of over 3 years from a patient in Berlin, Germany, with cystic fibrosis (CF); presence of the clone was not associated with disease. The genome sequences of three isolates of the same clone, including one isolate obtained from a different hospital in Germany, were used for comparative analysis of penicillin resistance determinants, the penicillin-binding proteins PBP2x, PBP1a, and PBP2b, and the main pneumococcal virulence factors.

## RESULTS

Twenty-nine *S. pneumoniae* isolates were obtained between 1992 and 1995 from the Wannsee-Lungenklinik-Heckeshorn in Berlin. Seven of these isolates were recovered from one CF patient over a period of 37 months and were not associated with disease (Table 1). All seven strains expressed serotype 23F and showed identical antibiotic resistance patterns that were distinct from patterns of the other 22 strains (data not shown). MLST revealed that these seven isolates were members of the same clone of a new ST, ST10523. Screening of our strain collection detected another member of ST10523, strain D219, which was isolated in Leipzig, Germany, in 1989 (25). In order to see whether special virulence factors are associated with this clone and whether the isolates from the CF patient differed from D219, the genomes of the first (D122) and last (D141) isolate from Berlin and of D219 from Jena were sequenced.

**TABLE 1:** Bacterial strains^a^

Species and isolate no.^b^	Date of isolation (day/mo/yr)	Site	ST	MIC (µg/ml)			TET/CLO/ERY susceptibility
				PEN-G	CTX	OXA	
*S. pneumoniae* (ST23F)							
D122	27/07/1992	Nasopharynx	10523	0.19–0.25	0.125–0.19	4–6	S/S/S
D127	8/4/1994	Nasopharynx	10523	0.19–0.25	0.125–0.19	4–6	S/S/S
D128	25/07/1994	Nasopharynx	10523	0.19–0.25	0.125–0.19	4–6	S/S/S
D134	31/10/1994	Nasopharynx	10523	0.19–0.25	0.125–0.19	4–6	S/S/S
D136	9/1/1995	Nasopharynx	10523	0.19–0.25	0.125–0.19	4–6	S/S/S
D139	9/5/1995	Sputum	10523	0.19–0.25	0.125–0.19	ND^d^	S/S/S
D141	1/8/1995	Nasopharynx	10523	0.19–0.25	0.125–0.19	4–6	S/S/S
D219	1989	Throat	10523	0.19–0.25	0.125–0.19	ND	S/S/S
*S. mitis*							
B8	1995	Oral cavity		0.12–0.2	0.006–0.03	0.5	S/S/S
B93-4^c^	1995	Oral cavity		ND	ND	ND	ND
B10	1995	Oral cavity		0.47	0.23	0.38	S/S/S
*S. oralis*							
B11	1995	Oral cavity		8	2	96	R/S/(R)

a S, sensitive; R, resistant; (R) intermediate resistant. Drug abbreviations: PEN-G, benzylpenicillin; CTX, cefotaxime; OXA, oxacillin; TET, tetracycline; CLO, chloramphenicol; ERY, erythromycin.

b All isolates were obtained from the Wannsee-Lungenklinik-Heckeshorn, Berlin, and from the same patient, except for D219, which was isolated in Leipzig (25).

c The strain could not be recovered after DNA isolation.

d ND, not determined.

### Antibiotic resistance and penicillin-binding proteins.

All seven ST10523 strains had intermediate resistance to beta-lactam antibiotics but were sensitive to tetracycline, erythromycin, and chloramphenicol (Table 1). Since PBP2x, PBP2b, and PBP1a play key roles in penicillin resistance, these genes were analyzed in detail to see whether they are related to PBP alleles of other penicillin-resistant *S. pneumoniae* (PRSP) strains. The PBP sequences of D219, D122, and D141 were identical and contained sequence blocks that diverged from PBP genes of the penicillin-sensitive R6 laboratory strain by ~20%. They did not match any other PBP sequences in the NCBI database except for *pbp1a* (see below). Interestingly, the genomic regions containing *pbp2x* (spr302 to spr307; *yllC* to *clpL*) and *pbp2b* (spr1513 to spr1517; *mutT* to *pbp2b*) contained a significantly larger amount of single nucleotide polymorphisms (SNPs; >5.3%) in the ST10523 strains than in the entire genome of the R6 strain, excluding variable genes (<0.7%), indicating transfer of large sequence blocks flanking the PBP genes. Similar observations have been reported previously (26). In contrast, flanking genes of *pbp1a* showed no signs of recombination events.

**FIG 1:**
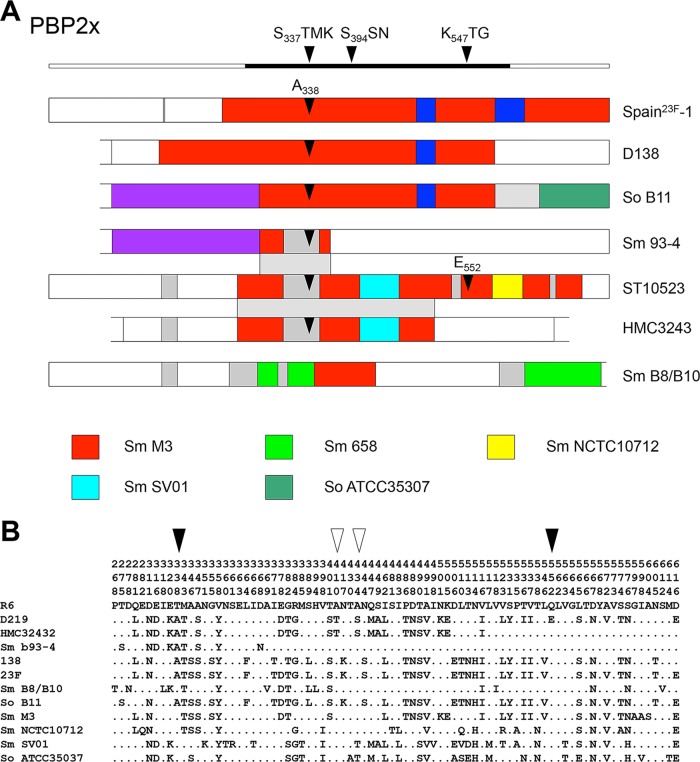
Schematic representation of the mosaic genes for PBP2x, PBP1a, and PBP2b from *S. pneumoniae* and *S. mitis* strains. (A) Mosaic sequence blocks in PBP2x. PBP2x genes of ST10523 (*S. pneumoniae* strains D122, D139, D141, and D219) are identical; *pbp2x* of *S. pneumoniae* Spain^23F^-1 is included for comparison. The *pbp2x* gene of *S. pneumoniae* HMC3243 from Romania, which is partially identical to *pbp2x* of ST10523, was also included. The colors indicate closely related sequence portions with >95% identity to *pbp2x* reference sequences from penicillin-sensitive *S. mitis* (Sm.) strains M3, 658, NCTC10712, and SV01 and *S. oralis* (So) ATCC 35307 (76), as indicated by the color coded boxes. White indicates sequences related to the penicillin-sensitive strain *S. pneumoniae* R6, and gray sequences are highly divergent from R6 sequences and other *pbp2x* reference sequences. The domain structure of PBP2x with the three homology boxes is shown above; the central penicillin-binding domain is indicated by the black bar. Black arrows represent mutations A_338_ and E_552_ in *pbp2x*. The regions that are identical between *pbp2x* of ST10523, *S. mitis* B93-4, and *S. pneumoniae* HMC3243 are indicated by the shaded area. (B) Deduced amino acid sequences of the transpeptidase domain of PBP2x; PBP2x of *S. pneumoniae* R6 was used as a reference. The positions of the amino acids are indicated vertically in the top three rows. Only positions with altered residues are shown; residues identical to those in strain R6 are indicated by dots. The mutations A_338_ and E_552_ are highlighted by black arrowheads; white arrowheads indicate amino acids which are not present in PBP2x of the penicillin-sensitive reference strains (see text for details).

The PBP2x gene of ST10523 has a complex mosaic structure (Fig. 1). It contains sequence blocks that are highly related (<3% difference on the DNA level) to *pbp2x* of putative penicillin-sensitive donor *S. mitis* strains M3, SV01, and NCTC10712 (27), in addition to other divergent sequences of unknown origin (Fig. 1A). The deduced protein sequence included mutations A_338_ and E_552_, which are known to confer resistance to beta-lactams; both amino acid changes are frequent in clinical PRSP isolates. The only other 2 amino acids that did not match any of the sensitive reference *S. mitis* strains were T_410_, present in *pbp2x* of low-level-resistant viridans streptococci, and T_434_, which occurs in some penicillin-sensitive strains (Fig. 1B) (27).

Furthermore, we obtained *pbp2x* sequences from four commensal streptococcus isolates from the same patient, isolates D122 and D141 (Table 1). The PBP2x genes of *S. mitis* strains B8 and B10 were identical and were distinct from *pbp2x* of *S. oralis* B11 and that of *S. pneumoniae* ST10523. The gene *pbp2x*_B11_ contained a central sequence block almost identical to that of Spain^23F^-1, and 3′-sequences were related to *S. oralis* ATCC 35307 (Fig. 1A). BLAST searches revealed one Romanian isolate, HMC3243, of unknown serotype (28) which was partially identical to D219 up to codon 517, whereas the C-terminal part represented R6 sequences (Fig. 1A and B). Interestingly, *pbp2x* of *S. mitis* B93-4 contained a 284-nucleotide (nt) sequence block almost identical to PBP2x_D219_ (codons 284 to 377), including two SNPs resulting in 1 amino acid change, D_368_N, suggesting that this block was acquired from *S. pneumoniae* ST10523 (Fig. 1A). The flanking 5′-sequence block was identical to *S. oralis* B11, and the 3′-end was identical to the *S. pneumoniae* R6 sequence, documenting multiple interspecies gene transfer events.

The internal mosaic blocks of ST10523 *pbp2b* and *pbp1a* were highly related to the respective genes in *S. mitis* NCTC10712 (see Fig. S1 in the supplemental material). PBP2b contained the mutation A_446_ close to the conserved S_443_SN motif, which mediates low resistance levels (29) and which is present in most penicillin-resistant isolates. Moreover, it had one more amino acid, Y_430_, that resulted in a deduced protein of 681 residues (Fig. S1). Regarding PBP1a of ST10523, the change of four consecutive residues, T_574_SQF to NTGY, has been associated with penicillin resistance, but the mutation A_371_ within the active site motif S_470_TMK, which also has been implicated in penicillin resistance, was not present (30–32). The PBP1a gene of ST10523 was identical to that of *S. pneumoniae* strain HMC3243 (Fig. S1) except for three silent SNPs. In contrast, the mosaic structure of *pbp2b* was entirely different. This clearly indicated that the three PBP genes were acquired from different sources or occasions in *S. pneumoniae* HMC3243 compared to ST10523. In summary, all three PBPs contained amino acid mutations that have been associated with the resistance phenotype corresponding to the intermediate penicillin resistance of ST10523.

10.1128/mSphere.00201-17.1 FIG S1  Mosaic structure of PBP1a and PBP2b of *S. pneumoniae* clone ST10523 and strain HMC3243. The homology boxes of PBP1a and PBP2b are shown above the mosaic genes. The mutation A446 in PBP2b is represented by the black arrow; the white arrow shows an additional amino acid. The colors indicate closely related sequences with >95% identity to the *pbp2x* reference sequences from penicillin-sensitive *S. mitis* strains M3 and NCTC10712, as indicated by the color coded boxes. Download FIG S1, TIF file, 6.6 MB. Copyright © 2017 Rieger et al. 2017 Rieger et al. This content is distributed under the terms of the Creative Commons Attribution 4.0 International license.

### Genomic comparison of *S. pneumoniae* genomes.

Since ST10523 is a new sequence type, the 2,050,063-nt draft genome of strain D219 was first compared to the R6 genome. Genes with no match in R6 were then used in a BLAST search of the NCBI database. Excluding transposases, the genome of D219 differed from the R6 genome by ~8%, including parts of a bacteriocin cluster (SPND219_00557 to SPND219_00567), the *cps* biosynthesis cluster encoding the 23F capsule (SPND219_00380 to SPND219_00398), and two clusters encoding phage-related genes (SPND219_00003 to SPND219_00023 [phage relict] and SPND219_01526 to SPND219_01585 [prophage]). This percentage corresponds to data obtained in comparative genomic hybridization on an oligonucleotide microarray representing the TIGR4 genome (33). Large parts of the phage relict were present in several genomes of *S. pneumoniae*. The prophage shows high similarity to *S. pneumoniae* phage 040922 (GenBank accession number FR671406), which is associated with a Tn*916*-like element in one *S. pneumoniae* strain, 18C/3 (34). The prophage contains two large genes (fragments SPND219_01501-3 and SPND219_01535) that encode the surface-expressed tail fiber PblB and the tape measure protein PblA. Homologues of PblA and PblB of *S. mitis* phage SM1 have been shown to be involved in the platelet-binding activity of *S. mitis* SF100 (35) and for its virulence in an animal model of infective endocarditis (36). No genes exclusively carried by ST10523 could be detected in BLAST searches.

Between the genome of D219 on one hand and D122 and D141 on the other hand, little difference regarding gene content was noted. The only exceptions were the phage relict and the prophage mentioned above, which were absent in D122 and D141. One gene cluster, SPND122_00705 to SPND122_00709 and SPND141_00707 to SPND141_00711 related to the R6 genes *spr0623* to *spr062*7 (ABC transporter, lactate monooxygenase, 2-lysyl-tRNA synthetase) were not found in D219. However, since these genes are all located on small contigs, including repeat elements such as BOX and RUP (37, 38), which result in problems during the genome assembly process, verification of their absence in D219 will require further analyses.

The genomes of D122 and D141 differed from each other by approximately 0.01% SNPs in 49 genes, less than that found in D219 (0.02 to 0.024% affecting a total of 178 genes in D219), i.e., the isolates from the patient are more closely related to each other than to D219, in agreement with their distinct place of isolation. One clear difference was observed in the gene encoding the two-component sensor kinase HK07 (39), which is not essential in *S. pneumoniae* (40). It was intact in strains D122 and D219 (SPND122_00180 and SPND219_00200), whereas an ISL3 family transposase fragment was inserted into the D141 gene, resulting in three incomplete gene fragments, SPND141_00182 to SPND141_00184.

### Virulence genes in ST10523.

All genes encoding the main *S. pneumoniae* specific virulence factors *lytA*, *ply*, *Nana*, and *nanB* and the CBPs *pspC*, *pspA*, and *pcpC* were present in the three ST10523 genomes. The deduced protein sequences were identical to each other and identical or highly similar to R6 proteins except for *pspA*, which represented a distinct genetic variant in ST10523. Differences in the repeat regions in genes encoding CBPs were not considered, since they are most likely caused by assembly problems of the choline-binding repeat regions. Other genes encoding surface proteins of important biological function, *pavA* and, for the IgA proteases, *zmpB* and *zmpA* were also present and identical in all three strains, except for the IgA protease, which contained gaps in the sequence of D219 and two SNPs plus a single-nucleotide deletion in D122, resulting in a premature stop codon. No pilus cluster was detectable. The capsule cluster SPND219_00380 to SPND219_00394 differed from that of *S. pneumoniae* strain Spain^23F^-1 by 20 SNPs, as expected for genes of distinct clonal origins. The last four-gene *rml* operon encoding enzymes involved in dTDP-rhamnose synthesis and which is common to several serotypes (41) included two short divergent regions in *rmlB* identical to *rmlB* of many serotype 19F strains.

**FIG 2:**
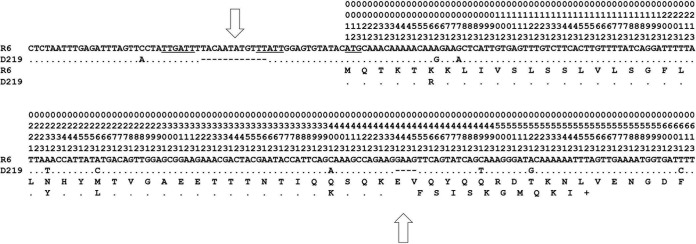
The HlyA gene of *S. pneumoniae* R6 and ST10523. The HlyA gene of *S. pneumoniae* R6, including upstream sequences (57 nt) to codon 72, is indicated. The −35 region, the −10 region, and the start codon ATG are underlined. The deduced amino acid sequence is indicated below. In the *hlyA* region of D219 (ST10523), only nucleotides and deduced amino acids that differ from the reference R6 sequence are shown. The 12-nt deletion upstream of the ATG start codon and the 4-nt deletion in the D219 sequence are indicated by open arrows. Vertical numbers in the first three rows refer to codons; numbers 1, 2, and 3 in the fourth row indicate the first, second, and third positions in the respective codon.

However, all three ST10523 genomes contained significant differences in *hlyA*, which encodes the hyaluronidase (SPND219_00348, SPND122_00329, and SPND141_00331) and which are unusual in other *S. pneumoniae* genomes. Within the HlyA gene, a 4-bp deletion corresponding to the position 128 nt downstream of the putative ATG start codon of R6 resulted in a premature stop codon. Moreover, a 12-nt deletion within the promoter region 10 nt upstream of the ATG start codon was present (Fig. 2). This strongly suggests that no functional hyaluronidase is expressed in ST10523 strains. Searches in the whole-genome contig NCBI database revealed another six genomes which contained this peculiarity (Table 2), but they differed by up to four SNPs from the D219 region shown in Fig. 2.

**TABLE 2:** *S. pneumoniae* genome sequences with an incomplete *hlyA*

Accession no.	Strain	Serotype	Source	Date (day/mo/yr)	Country (region or city)
LJVO01000185	NTPn 4	NT	Blood	2004	South Africa (KwaZulu-Natal)
CVHP01000011	0338	NT	Blood	2001	USA (Alaska)
CKDL01000005	Type strain	NT	Nasopharynx	14/4/2008	Thailand (Maela)
CPLS01000001	LMG205	6B	Not known	2008	Thailand
CRPU01000001	SMRU824	NT	Nasopharynx	10/10/2008	Thailand (Maela)
AGOE01000004	GA16531	NA^a^	NA	2001	USA (metropolitan Atlanta)
CFNW01000018	6378-99	19F	Not known	1999	USA (Tennessee)

a NA, not available.

## DISCUSSION

Prolonged carriage of the same *S. pneumoniae* clone for a period of 37 months, as observed for serotype 23F isolates obtained from a CF patient in our study, is unusual. *S. pneumoniae* is not considered a persistent colonizer in CF patients, unlike *Pseudomonas aeruginosa* and *Staphylococcus aureus* (42–44). Long-term persistence has been reported for *Staphylococcus aureus* for up to 70 months (45). The duration of pneumococcal carriage in healthy children is a few weeks, ranging from 2 days and in rare cases up to 6 to 12 months, depending on the age of the carrier and the serotype of the isolate (2, 3, 46–48). Some serogroups, including serotype 23F, are generally carried for longer periods than other serogroups (46, 49) and have a low propensity to cause invasive disease (50). Interestingly, an inverse relationship between the attack rate of a given capsular serotype and its duration of carriage has been noted (3).

Carriage rates for *S. pneumoniae* isolated from CF patients are similar, with colonization rates ranging between 3 and 20% (51–56). No special serotypes appear to be associated with CF (57), but some serotypes may be more common, depending on the geographic area. Serotype 23F isolates were prevalent in a CF unit in Madrid, mainly due to the clone Spain^23F^-1 of a varied multiresistance phenotype (52). Serotype 3 prevailed in another study which reported no 23F serotype isolates in a CF center in Rome, probably because all patients had received vaccination (58). In both studies, *S. pneumoniae* was recovered more than once from some patients. However, only three strains of serotype 23F isolated over a period of 3 months showed identical SmaI restriction patterns, revealed by pulsed-field gel electrophoresis (PFGE), and the same antibiotic resistance profile, and thus most likely represented members of the same clone (52). Three patients carried *S. pneumoniae* with the same serotype and identical SmaI PFGE pattern for 1 to 8 months (58). In these cases, *S. pneumoniae* was considered a colonizer, since at the time of isolation the patients showed no evidence of pulmonary exacerbation.

Genomic comparisons showed that the two strains, D122 and D141, from the CF patient are more closely related to each other than to D219, which was isolated 3 years before from a different geographical site. They differed from D219 by the absence of two large gene clusters encoding a prophage and a phage relict, by the presence of a five-gene cluster, including an amino acid ABC transporter, and by the estimated number of SNPs. The prophage carries two genes encoding large proteins PblA and PblB. Homologues of these proteins are frequent in *S. pneumoniae* phages (59) and have been shown to play a role in adhesion and virulence in *S. mitis* (35, 36). It these proteins play a similar role in *S. pneumoniae*, it is conceivable that their absence in D122 and D141 supports extended carriage.

The variation between D122 and D141 concerned only 49 genes, and D141 contained an insertion of an ISL3 family transposase fragment into the gene encoding the histidine protein kinase HK07, which was absent in the D122 gene. This element was present at another three sites in the D141 genome and at one site in the D122 genome, whereas it could not be detected in D219. Other studies have supported little genomic variation during carriage of the same *S. pneumoniae* clone. Minimal variation was observed during carriage established experimentally with a single serotype 6B strain of ST138 (60). The maximum SNP distance between any of the 229 isolates obtained over a period of 35 days versus the reference strain was three SNPs (60). It should be noted that the genomic comparison between two isolates of strain D39, a historically important serotype 2 isolate from the early 1940s (61) and which have been cultivated separately for at least 21 years, revealed only five mutations (62). Similarly, some strains isolated during a 7-month period from a child with chronic pneumococcal infection varied by only ≤30 SNPs (63). However, those authors noted there were also multiple events of horizontal gene transfer in some strains, which most likely occurred during polyclonal infection. In contrast, we saw no evidence of gene acquisition in D141 versus D122, and the *S. mitis* strain B93-4 contained a *pbp2x* fragment identical to *pbp2x* of ST10523. The mosaic PBP2x and PBP2b genes of ST10523 represent new gene variants and are distinct from all others found in the NCBI database. Therefore, this finding indicates interspecies gene transfer from *S. pneumoniae* to *S. mitis* in the same host.

No genes specifically associated with ST10523 genomes were identified. This is not astounding, given the vast number of actually available genomes of *S. pneumoniae*. Based on a pan-genome analysis of 158 *S. pneumoniae* genomes, it has been predicted that only 0.3 new genes will be discovered in a new genome if a data set from 1,000 genomes is already available, and only 0.06 new genes will be discovered from 5,000 genomes (24). However, an unusual hyaluronidase gene, *hlyA*, was present in the ST10523 genomes which contained a stop codon (Fig. 2) distinct from that described in a serotype 3 clone, ST180, where an SNP at position 376 of the hyaluronidase coding sequence resulted in a stop codon and truncation of the protein after 125 amino acids (24). Moreover, a 12-nt deletion within the promoter region 10 nt upstream of the ATG was detected. Hyaluronidase is produced by almost all clinical isolates of pneumococci (64). It is one of the genes which have not been found in closely related viridans streptococci, except for some *S. oralis* isolates, but not in *S. mitis* strains (65), i.e., it represents a typical component of the species *S. pneumoniae*. The enzyme depolymerizes hyaluronic acid, which is an important component of the host connective tissue and extracellular matrix (66). No significant impact on virulence was found in a mouse intraperitoneal infection model when a single *hlyA* mutant was used (67); however, when a double mutant that was also deficient for the pneumolysin gene *ply* was tested, virulence was significantly decreased (68). Hyaluronidase significantly potentiated pneumolysin-mediated ciliary slowing and epithelial damage in an *in vitro* model, suggesting that its presence favors colonization and subsequently extrapulmonary dissemination of the pneumococcus (69). It is therefore tempting to assume that the lack of a functional hyaluronidase predestines ST10523 to survive within the host for an extended period without causing disease. In conclusion, the unusually long carriage rate observed for *S. pneumoniae* isolates D122 and D141 might not be related to mutations or genetic variants acquired during persistence in the human host, but rather to the loss of a large prophage carrying potential virulence factors and to the absence of a complete hyaluronidase gene.

## MATERIALS AND METHODS

### Bacterial strains and growth conditions.

*S. pneumoniae* strains D122 to D141, *S. mitis* B8, B93-4, and B10, and *S. oralis* B11 (Table 1) were part of a strain collection obtained from the Wannsee-Lungenklinik-Heckeshorn in Berlin; strain D219, isolated in Leipzig, has been described elsewhere (25). Strains were grown at 37°C without aeration in complex C medium (70) supplemented with 0.1% yeast extract (C + Y). MICs were determined by the agar dilution method (for beta-lactams) or by using E-test strips for all other antibiotics (Oxoid GmbH, Basingstoke, United Kingdom) on d-agar plates supplemented with 3% sheep blood (71).

### DNA sequencing and analysis.

Chromosomal DNA from streptococci was isolated as described previously (72). Internal sequences of the seven housekeeping genes were obtained with primers described on the *Streptococcus pneumoniae* MLST homepage (https://pubmlst.org/spneumoniae). PBP2x gene fragments were amplified with the primers pn2xup and pn2xdown (72), and direct sequencing of PCR products was performed with consecutive primers. PCR products were purified using a JetQuick DNA purification kit (GenoMed). PCRs were performed using either Goldstar Red *Taq* polymerase (Eurogentec) or DreamTaq polymerase (Fermentas), according to the manufacturer instructions. The genomes of D219, D122, and D144 were sequenced using a 454 Life Sciences FLX sequencer, and reads were assembled by the 454 Newbler Assembler version 2.6. Contigs were aligned to the *S. pneumoniae* R6 genome sequence (73). The rapid annotation subsystem technology (RAST) server (74) designed for annotation of bacterial and archaeal genomes was applied to obtain EMBL-formatted files containing protein, tRNA, and rRNA annotations from a large set of several output formats.

### DNA analysis and bioinformatic tools.

For the analysis of SNPs in the three ST10523 genomes, only sequences that were 350 nt from contig ends were included, to avoid potential errors generated by the 454-generated sequences. Individual open reading frames were investigated manually and compared with other genome sequences by using BLAST analyses and the NCBI database (nucleotides and whole-genome contigs). As a reference for the serotype 23F capsule cluster, genes of strain ATCC (Spain^23F^-1) were used (75). Alignments were prepared using Clustal X2 (76). Codon sites were included manually and trimmed by using the program Clustal Formatter 3 (http://nbc11.biologie.uni-kl.de/sequence_analysis/ClustalFormatter3/documentation.html) to reveal only sites that differ from the reference sequence shown in Fig. 1B.

### Accession number(s).

The following sequences have been submitted to GenBank and assigned the following accession numbers (shown in parentheses): for genomes, D219 (CP016227), D122 (CP016632), D141 (CP016633); for PBP genes of *S. pneumoniae* strain HMC3243, *pbp2x* (FJ439546), *pbp1a* (FJ439538), and *pbp2b* (FJ439554) (28); for PBP2x genes, *S. mitis* strain B8 (KY292528), strain SV01 (KY292540), and strain B93-4 (KY783589), and *S. oralis* strain B11 (KY783587).
